# Quantitation of nucleoprotein complexes by UV absorbance and Bradford assay

**DOI:** 10.52601/bpr.2021.210028

**Published:** 2021-12-31

**Authors:** Jiang Chen, Hao Luo, Mei Tao, Zhongchuan Liu, Ganggang Wang

**Affiliations:** 1 Key Laboratory of Environmental and Applied Microbiology, Chengdu Institute of Biology, Chinese Academy of Sciences; Key Laboratory of Environmental Microbiology of Sichuan Province, Chengdu 610041, China; 2 College of Life Sciences, Sichuan University, Chengdu 610064, China; 3 University of Chinese Academy of Sciences, Beijing 100049, China

**Keywords:** Nucleoprotein complex, Quantitation, Bradford assay, UV-derived formula-based method

## Abstract

Despite the importance of studying nucleoprotein complexes, no appropriate method for quantifying them is available. Here, a UV absorbance method using the formula “*C*_mg/mL_ = 1.55*A*_280_ – 0.76*A*_260_” were applied to quantify nucleoprotein complexes. After modification using two paired A_260_ and A_280_ values, the UV-derived formula-based method could accurately quantify proteins in nucleoprotein complexes. Otherwise, by taking the target protein as a standard, the Bradford assay can accurately quantify proteins in nucleoprotein complexes without interference by nucleic acids. The above methods were successfully applied to measure the concentration of *Mtu*P49-CTG complexes of* Mycobacterium tuberculosis*. In conclusion, both the Bradford assay and the UV-derived formula-based method were appropriate for quantifying proteins in nucleoprotein complexes, which may make contributions to explore the interactions between proteins and nucleic acids at the molecular level.

## INTRODUCTION

Interactions between nucleic acids and proteins are at the heart of the field of cellular information processing, such as DNA replication, DNA transcription, DNA translation, and post-translational regulation (Gao *et al*. 2019; Spies and Smith 2017). Therefore, exploring the interactions between proteins and nucleic acids is of particular interest in the fields of biology, clinical medicine, and chemistry (Gao *et al*. 2019; Gardner and Kelman 2019). Methodological advances have now made it possible to comprehensively investigate such interactions by biochemical and biophysical approaches (Kuznetsova *et al*. 2016; Lin and Wu 2019), which usually acquire accurate quantitation of nucleoprotein complex.

The accurate concentration of nucleoprotein complexes can be reflected by quantifying its protein components. Protein concentrations can be measured by UV absorbance methods dye-binding assays (Noble 2014). However, the overlap in the typical absorption of protein and nucleic acid makes it difficult to quantify nucleoprotein complexes directly by the UV absorbance methods. Although several methods have been developed to determine the content of protein when binding or mixing with nucleic acid (Groves *et al*. 1968; Porterfield and Zlotnick 2010), they were not widely applied into practice, due to the relatively laborious process or the deviation of estimated ε (Burgess *et al.* 2009; Sanchez 2021). Therefore, a rapid and self-consistent method for determining the concentrations of proteins in nucleoprotein complexes was required.

Based on A_280_ and A_260_, an empirical formula was established to estimate the protein content when mixed with nucleic acid:

1 $ C_{{\rm{mg/mL}}} = 1.55A_{280} - 0.76A_{260}, $

where *A*_280_ and *A*_260_ represent the UV absorption at 280 nm and 260 nm with 1 cm of optical path length (Goldring 2015). By this formula, the concentration of proteins can be directly calculated without the need for a standard, which enables to measure the protein concentration more quickly. However, this method has not been widely used in practice and the accuracy of this formula is therefore unknown.

As for dye-binding assays, the most widely used method was the Bradford assay (Noble 2014), which requires a standard curve to calculate the concentration of an unknown protein (Noble 2014). To our knowledge, no studies have reported whether this method is suitable for the quantitative analysis of nucleoprotein complexes.

In this study, the UV absorbance method based on Eq. 1 and the Bradford assay was modified and applied to quantify proteins concentrations in nucleoprotein complexes. Here, three nucleoprotein complexes were studied, namely, the nonspecific binding between *Bacillus stearothermophilus* DnaB (*Bst*DnaB) and 5’-TTTTTTTTTTTTTTTT-3’ (dT16) (Bird and Wigley 1999), the specific binding between *Bacillus subtilis* DnaG (*Bsu*DnaG) and 5’-CAGACACACACACTACACACA-3’ (CTA) (Zhou *et al*. 2017), and the specific binding between *Saccharomyces cerevisiae* forkhead homolog DNA-binding domain (Fhk1-DBD) and a complementary double-stranded DNA (dsDNA) (Hollenhorst *et al*. 2001), with lysozyme and BSA mixed with CTA or dsDNA were used as controls. The results showed that it is feasible to quantify protein concentration in nucleoprotein complexes by both the Bradford assay and UV absorbance method.

## MATERIALS AND METHODS

### Reagents and proteins

Tris (hydroxymethyl) methyl aminomethane (Tris, A501492-0005), dithiothreitol (DTT, A300862-0005), Coomassie brilliant blue G250 (6104-58-1) were purchased from Sangon Biotech (Shanghai, CHN). Glycerol and MgCl_2_ were purchased from Chron Chemicals (Chengdu, CHN). Ultra-pure water was obtained using a Milli-Q system from Merck Millipore (San Diego, CA, USA).

**Figure 1:**
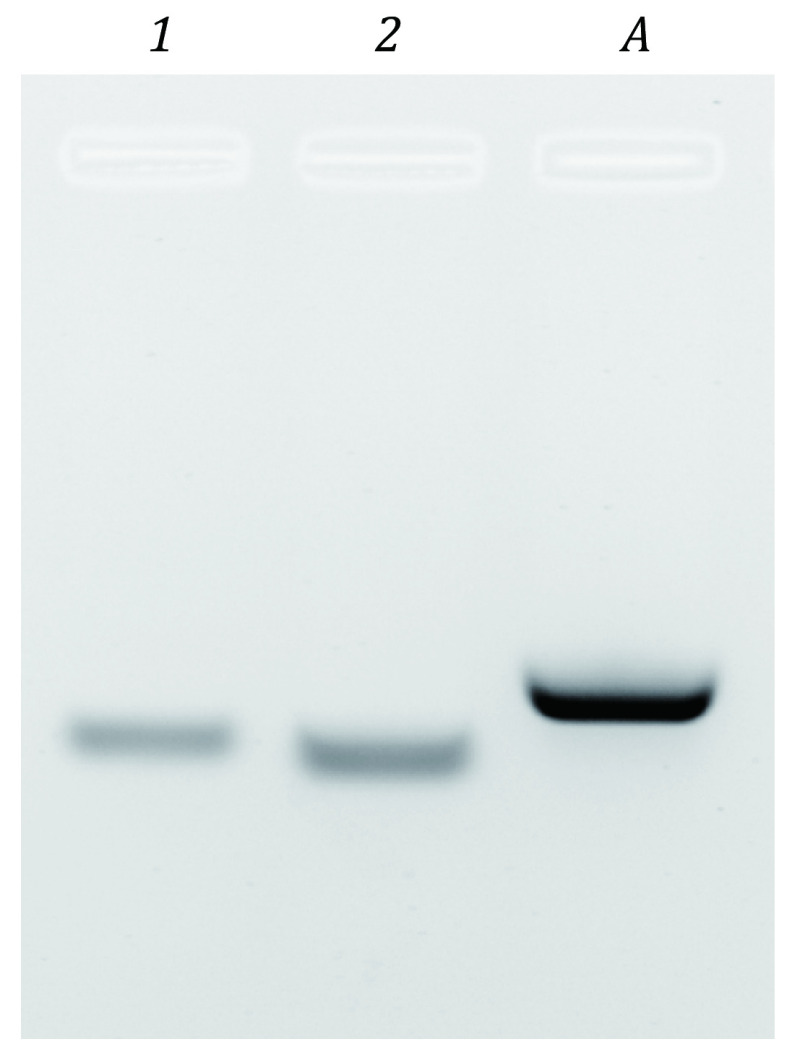
Annealing of ssDNAs to create dsDNA ligand for Fhk1-DBD. ~300 ng of each indicated ss/dsDNA was run on a 3.5% agarose gel at 25 V/cm for 20 min in 0.5 × TB to distinguish the ssDNA 22-mers from the annealed dsDNA complex. *1*: 5’-TGCAAAATGTAAACAAGACT-3’; *2*: 5’-AGTCTTGTTTACATTTTGCA-3’; *A*: the annealed dsDNA

Lysozyme was purchased from Solarbio (CHN; No. 1114H046, 14.0–15.0 kDa); BSA was purchased from Roche (CHE; No. 10735078001, 66.0 kDa); *Bacillus stearothermophilus* DnaB (*Bst*DnaB, 50.6 kDa), *Bacillus subtilis* DnaG (*Bsu*DnaG, 68.7 kDa), and *Saccharomyces cerevisiae* forkhead homolog DNA-binding domain (Fhk1-DBD, 16.0 kDa) were purified as described previously (Yang and Wang 2016; Zhou *et al*. 2017), which were stored at −80 °C before use. Oligo DNA was synthesized by Sangon Biotech. The two complementary ssDNAs (5’-TGCAAAATGTAAACAAGACT-3’ and 5’-AGTCTTGTTTACATTTTGCA-3’) were annealed by mixing equivalent molar amounts of each ssDNA in an annealing buffer (10 mmol/L Tris-HCl, pH 8.0, 50 mmol/L NaCl, and 1 mmol/L EDTA), heating to 95 °C for 5 min, and then allowing the solution to slowly cool to room temperature over 30 min to form dsDNA. Successful annealing was confirmed by agarose gel electrophoresis (Fig. 1).

### Quantitation of protein using the equation *C*_mg/mL _= 1.55*A*_280_ – 0.76*A*_260_

The proteins and nucleic acids were dissolved/diluted to a certain concentration in a binding buffer (25 mmol/L Tris-HCl, pH 8.0, 100 mmol/L NaCl, 1 mmol/L DTT, 10 mmol/L MgCl_2_, and 5% glycerol), centrifuged at 4 °C, 12,000 *g* for 20 min, and stored in an ice bath. To prepare nucleoprotein complexes/mixtures with a series of molar ratios on nucleic acid / protein (N/Ps), 5 μL of protein was mixed with 0–10 μL of corresponding nucleic acid, which was added to 15 μL by the binding buffer. After binding at 25 °C for 30 min, A_260_ and A_280_ of the samples were collected by a NanoDrop Lite under the nucleic acid detection mode, with binding buffer as a blank (Desjardins and Conklin 2010). After that, the paired A_260_ and A_280_ values were applied to quantify the proteins of samples using the empirical formula (Eq. 1).

### Modification of formula to improve the quantitation accuracy

To modify and refine the empirical formula, essentially to revise the two coefficients *K*_280_ and *K*_260_, at least two paired A_260_ and A_280_ were needed, to prepare the followed two quadratic equations:

2 $ C_{{\rm{mg/mL}}} = (K_{280} \cdot A_{280}^{1}) - (K_{260} \cdot A_{260}^{1}), $

3 $ C_{{\rm{mg/mL}}} = (K_{280} \cdot A_{280}^{2}) - (K_{260} \cdot A_{260}^{2}),   $

where *A*_280_^1^, *A*_280_^2^ and *A*_260_^1^, *A*_260_^2^ represent the UV absorption at 280 nm and 260 nm of Samples 1 and 2 with different molar ratios of nucleic acids, as mentioned above. According to the sample preparation, the protein concentrations of all samples with different N/Ps were constant, so *C*_mg/mL_, *i*.*e*., the concentration of protein was known, the modified coefficients can then be obtained by solving the simultaneous Eq. 2 and Eq. 3. As a result, modified formulas were acquired, which were then applied to quantify the corresponding protein concentrations, using the series of paired A_260_ and A_280_ values above.

### Bradford assay for protein quantitation

To prepare the dyeing solution, 50 mg of Coomassie brilliant blue G250 was first dissolved in 25 mL of 95% ethanol, followed by mixing with 50 mL of 85% H_3_PO_4_. After adjusting to 500 mL with ddH_2_O, the dyeing solution was filtrated with a 0.22 μmol/L filter and stored at room temperature before use. The samples with a series of N/Ps were prepared by the method mentioned above. For dyeing, 5 μL samples were mixed with 45 μL of binding buffer in a 96-well plate. After thoroughly mixing, 200 μL of dyeing solution was added to the mixture, which was then lightly shaken for 20 min. The absorbances at 595 nm of the samples were collected by a Varioskan Flash (Thermo Fisher Scientific). The protein concentrations were calculated and expressed as mg/mL based on the standard curves prepared with lysozyme (0.22–1.1 mg/mL), BSA (0.194–0.97 mg/mL), *Bst*DnaB (0.22–1.1 mg/mL), *Bsu*DnaG (0.22–1.1 mg/mL), and Fhk1-DBD (0.22–1.1 mg/mL).

### Method validation using *Mycobacterium tuberculosis (Mt)* DnaG–CTG complexes

**Figure 2:**
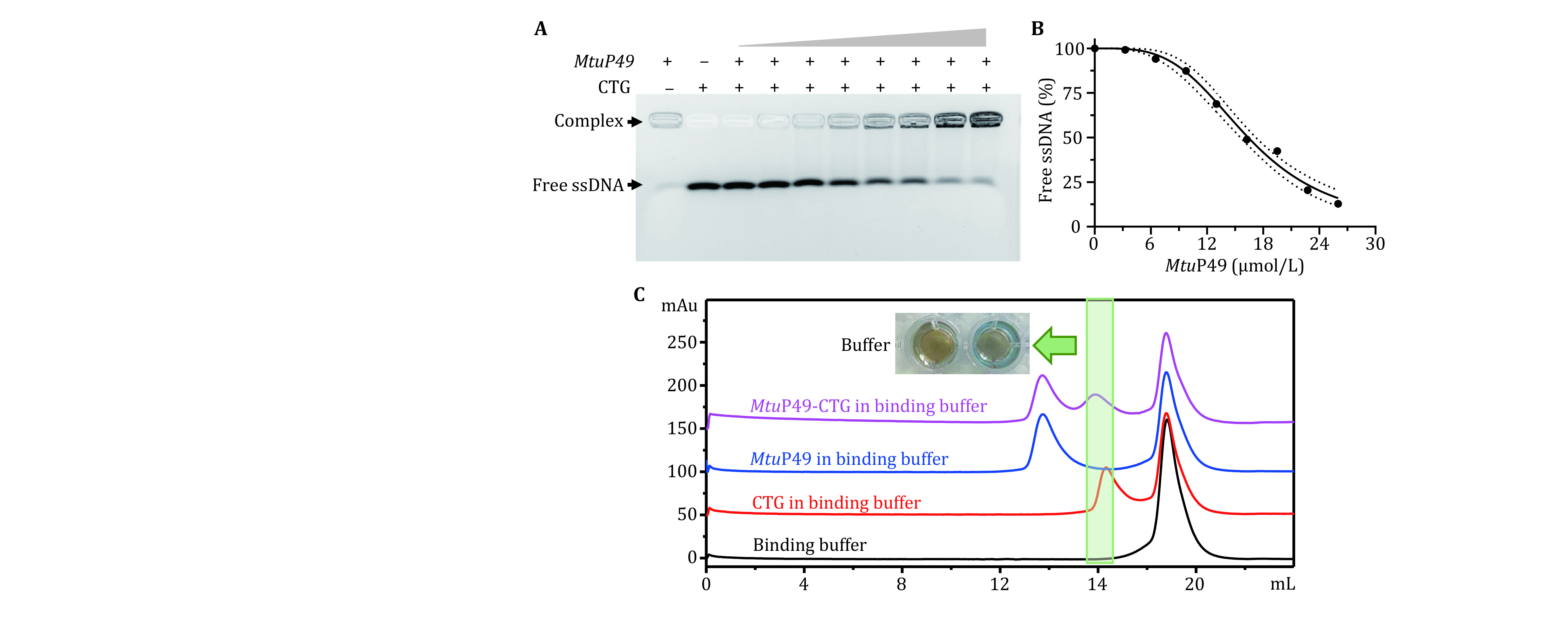
Preparation of MtuP49-CTG complex. **A** MtuP49 was mixed with a gradient concentration of CTG, and the samples were run on a 3.0% UltraGelRed predyeing-agarose gel at 20 V/cm for 20 min in 0.5× TB. And the results showed that the content of free nucleic acid decreased gradually, with the increasing concentration of MtuP49, and more and more complexes were formed. **B** Binding curve of MtuP49-CTG by free ssDNA, using the ImageJ. **C** purification of MtuP49-CTG complex by Superdex 200 increase gel filtration column (GE Healthcare, 10 × 300). By this gel filtration column, the complex can be well separated from MtuP49, while its retention time was close with a nucleic acid. For this reason, the excess MtuP49 was added to completely bind the free CTG

To confirm the feasibility of the above method, *Mt*DnaG ZBD−RPD domain (*Mtu*P49) was prepared, which was expressed in *Escherichia coli* BL21(DE3) cells by connecting with pGEX-6P-1 and sequentially purified by a method described previously (Zhou *et al*. 2017). In the follow-up study, *Mtu*P49 was shown to interact with the ssDNA CTG (Fig. 2A and 2B). In this study, excess *Mtu*P49 was mixed with CTG and incubated at 30 °C for 30 min in a binding buffer (PBS, pH 6.5, 100 mmol/L NaCl, 10% glycerol, 5 mmol/L MgCl_2_, 1 mmol/L ATP, 2 mmol/L DTT). The complexes were purified using a Superdex 200 increase gel filtration column (GE Healthcare, 10 × 300) with an elution buffer (PBS, pH 6.5, 100 mmol/L NaCl, 10% glycerol, 2 mmol/L DTT) (Fig. 2C). After ultrafiltration, the concentration of protein in the *Mtu*P49/ssDNA complexes was then acquired by both Bradford assay (using *Mtu*P49 as a standard) and the modified formula, which was modified by two paired A_280_ and A_260_ values of samples with N/Ps of 0/1 and 3/1 were obtained as mentioned above, with ~3 mg/mL *Mtu*P49.

### Statistical analysis

All data are expressed as mean ± SD from triplicate experiments. Statistical analysis and plotting were performed using GraphPad Prism 8.2.1 (San Diego, CA, USA). Significant differences were evaluated by unpaired *t*-test, by the Holm–Sidak method, with an α-value under 0.05 being considered to represent a statistically significant difference.

## RESULTS

### Protein quantitation using empirical formula was unsatisfactory

**Figure 3:**
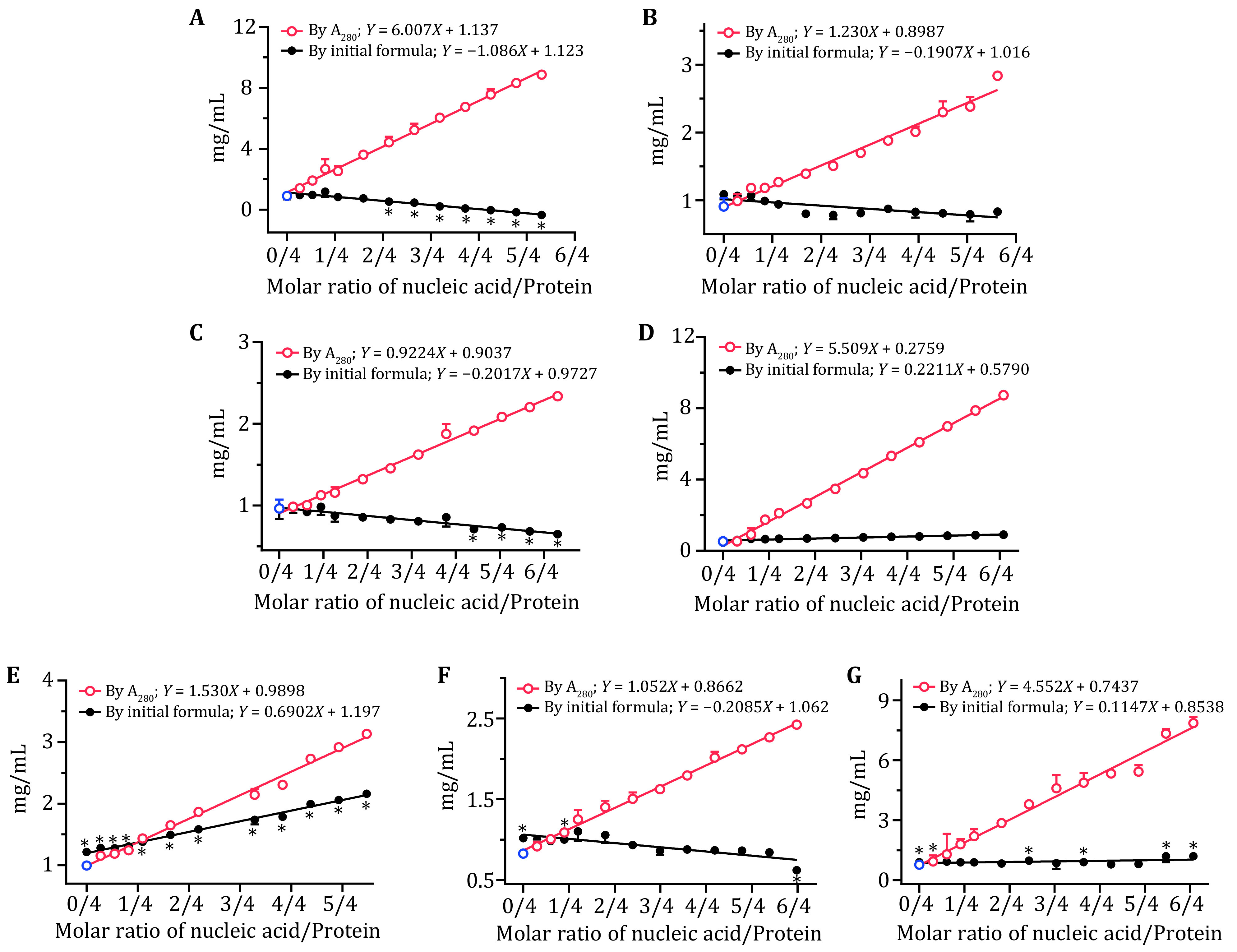
Protein quantitation by the empirical formula. **A** BSA–CTA mixture. **B** BSA–dsDNA mixture. **C** Lysozyme–CTA mixture. **D** Lysozyme–dsDNA mixture. **E** *Bst*DnaB–dT16 complex/mixture. **F** *Bsu*DnaG–CTA complex/mixture. **G** Fkh1–DBD–dsDNA complex/mixture. The protein concentrations among the complexes were first measured by Nanodrop, based on A_280_, as marked as red circles, where the blue circles represent the actual concentrations with no nucleic acid added. The black dots represent the quantified protein concentrations among the above groups, as calculated using the empirical formula (Eq. 1), based on A_280_ and A_260_. Unpaired *t*-test by the Holm-Sidak method was used to analyze statistical difference, with * for *p* < 0.05

Samples with the series of molar ratios on nucleic acid / protein (N/Ps) were prepared. After binding, A_260_ and A_280_ of the samples were simultaneously collected. As showed in Fig. 3 (the red lines), A_280_-originated protein concentrations increased linearly along with the addition of nucleic acids, which confirms that the quantitation of proteins in nucleoprotein complexes directly using A_280_ was extremely inaccurate. And then, the empirical formula (Eq. 1) was tentatively applied to correct it, using the paired A_260_ and A_280_ values collected above.

Using the empirical formula (Eq. 1), the error caused by nucleic acid can be dramatically reduced and the protein concentrations calculated were much closer to the actual ones (Fig. 3). Actually, the quantified concentrations showed no significant difference from the actual ones for lysozyme–dsDNA and Fkh1–DBD–dsDNA (Fig. 3D and 3G), within the designed N/P ranges, while the calculated concentrations for other samples still deviated from the actual ones, especially for *Bst*DnaB–dT16 (Fig. 3E). Clearly, the empirical formula (Eq. 1) is helpful to reduce error for protein quantitation, while it cannot be applied for accurately quantifying proteins in nucleoprotein complexes.

### Modification of the empirical formula

It was found that there was a good linear relationship between the corrected protein concentration and the N/Ps (Fig. 3), which makes it possible to modify the formula to improve its effectiveness. In theory, a straight line can be made by at least two points, which can be acquired from two paired A_260_ and A_280_ values of nucleoprotein complexes. As shown in Fig. 3, since the actual concentration of protein was known, the *K*_260_ and *K*_280_ in the formula (Eq. 1) can be revised with two paired A_260_ and A_280_, then a modified formula will be obtained.

Here, we took *Bst*DnaB–dT16 as an example, to elaborate the modification process in detail. As shown in Fig. 3E, protein quantitation using the original formula (Eq. 1) was unsatisfactory, since all calculated concentrations showed a significant difference from the actual one (0.996 mg/mL, *p* < 0.05). Initially, two paired values of A_260_ and A_280_, at N/Ps of 0 and 0.2737, were randomly selected for the modification, two binary linear equations were acquired.

For N/Ps of 0:

4 $ \;0.996 = 0.996K_{280} - 0.432K_{260}; $

For N/Ps of 0.2737:

5 $ \;0.996 = 1.434K_{280} - 1.107K_{260} . $

By solving the two equations Eq. 4 and Eq. 5, *K*_280_ and *K*_260_ were acquired as *K*_280_ = 1.393 and *K*_260_ = 0.906, and the empirical formula (Eq. 1) was hence modified as:

6 $ C_{{\rm{mg/mL}}} = 1.393A_{280} - 0.906A_{260}. $

**Figure 4:**
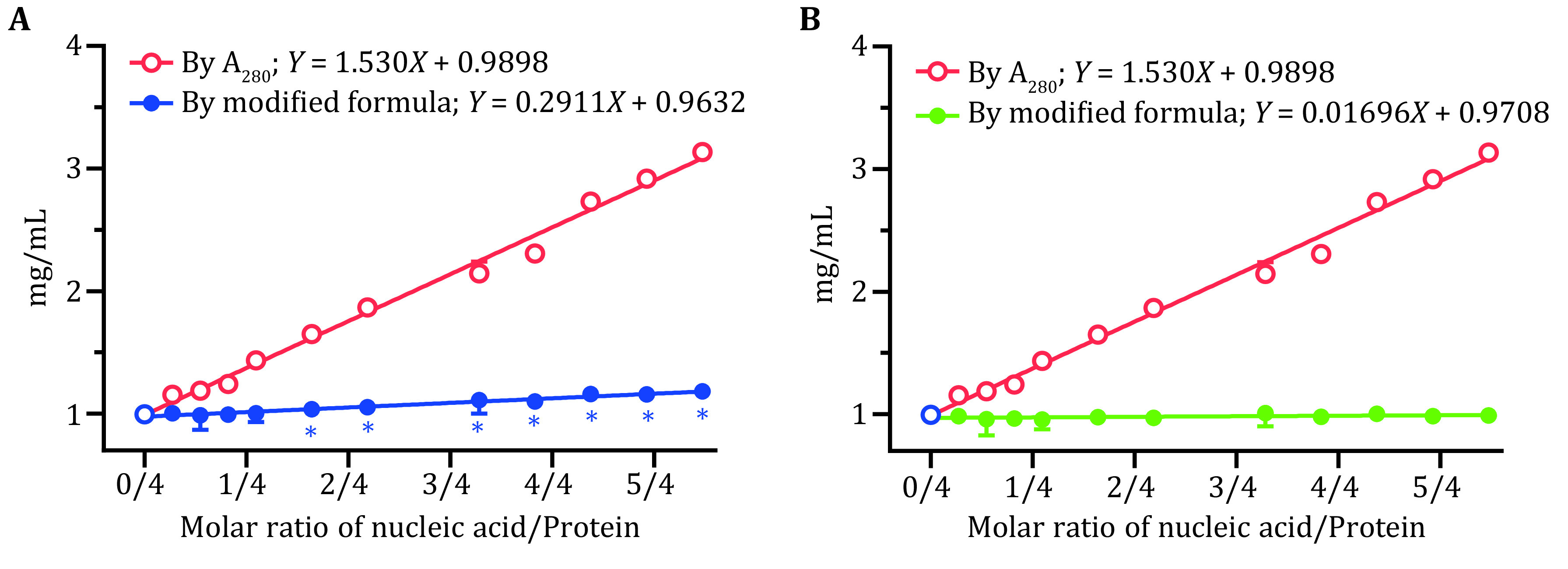
Protein quantitation of *Bst*DnaB–dT16 by modified formula. **A** Protein concentration (blue line) calculated using the formula (Eq. 6), which was modified from the empirical formula (Eq. 1) with paired values of A_280_ and A_260_ corresponding to N/Ps of 0 and 0.2737. **B** Protein concentration (green line) calculated using the formula (Eq. 8), which was modified from the empirical formula (Eq. 1) with paired values of A_280_ and A_260_ corresponding to N/Ps of 0 and1.3687. The protein concentrations among *Bst*DnaB–dT16 complexes measured by Nanodrop, based on A_280_, were marked as red circles; the blue circles represented the actual concentrations with no nucleic acid added. Unpaired *t*-test by the Holm-Sidak method was used to analyze statistical difference, with * for *p* < 0.05

Using the modified formula (Eq. 6), the protein concentration in *Bst*DnaB–dT16 complexes was requantified and plotted in Fig. 4A as a blue line. It seemed that the quantitation ability of the formula was notably improved, there was no significant difference between the requantified protein concentration and the actual one (*p* > 0.05) within N/Ps of ~0/4–0.5/4. In addition, the requantified protein concentrations were also linearly fitted to the N/Ps values, and a linear equation was obtained as *Y* = 0.2911*X* + 0.9632. Here, the slope is much gentler than the initial one, which again indicates that the quantitation ability of the formula was notably improved. However, the slope here still deviated significantly from 0 (*p* < 0.05), which means that the requantified protein concentration would also increase with the addition of nucleic acid, and the quantitation ability of the modified formula was still not ideal.

The above results seemingly implied that the range of selected data may determine the scope of application of the modified formula. To test this, we chose the paired A_260_ and A_280_ values with N/Ps of 0 and 1.3687, the minimal and maximal molar ratios applied here, to modify the formula again.

For N/Ps of 1.3687:

7 $ 1 = 3.135K_{280} - 3.551K_{260}.  $

In addition, *K*_280_ and *K*_260_ can be acquired by solving the two equations (Eq. 4) and (Eq. 7), as *K*_280_ = 1.424 and *K*_260_ = 0.977, and the empirical formula was then modified again as:

8 $ C_{{\rm{mg/mL}}} = 1.424A_{280} - 0.977A_{260}. $

Using the modified formula (Eq. 8), the protein concentration of *Bst*DnaB–dT16 was requantified and plotted in Fig. 4B as a green line. Surprisingly, there was no significant difference between all requantified protein concentrations and the actual one (*p* > 0.05), which indicates that using the modified formula (Eq. 8) can accurately quantify protein concentration in *Bst*DnaB–dT16 complexes, as also illustrated by the slope of the linear equation (*Y* = 0.01696*X* + 0.9708) fitted between the recorrected protein concentrations and N/Ps, which did not deviate significantly from 0 (*p* > 0.05).

In the same way, the empirical formula (Eq. 1) was modified with the paired A_260_ and A_280_ values corresponding to the minimal and maximal N/Ps of other nucleoprotein complexes/mixtures mentioned above. The modified formulas are presented in supplementary Table S1, while the requantified protein concentrations are shown in supplementary Fig. S1.

### Quantitation of nucleoprotein complexes by Bradford assay

The Bradford assay is commonly applied for the quantitative analysis of proteins. To our knowledge, no studies have reported on the quantitation of nucleoprotein complexes by this method. Here, the protein concentrations of samples with the series of N/Ps were measured by this assay. As shown in supplementary Fig. S2, the protein concentration was consistent with the increase of N/Ps values, which was confirmed by the gentle slopes of linear regression equations generated between the calculated protein concentration and the actual one. The incubation of different nucleic acids with Coomassie brilliant blue dye also showed no obvious color reaction (supplementary Fig. S3). Therefore, the Bradford assay is suitable for quantifying the proteins in nucleoprotein complexes.

However, the selection of standards has a relatively large impact on the results. Conventionally, BSA was used as the standard for the Bradford assay, but the data showed that the protein concentrations calculated with BSA mostly deviated from the actual concentration (supplementary Fig. S2). The lysozyme concentrations calculated using BSA as a standard were significantly lower than those with the lysozyme as a standard (supplementary Fig. S2C and S2D, *p* < 0.05), while the *Bst*DnaB concentration calculated using the BSA standard was significantly higher than those with the *Bst*DnaB standard (supplementary Fig. S2E, *p* < 0.05). Therefore, to obtain more accurate results, target protein should be taken as the standard.

### Method validation using *Mtu*P49–CTG complexes

The concentration of *Mtu*P49–CTG was determined simultaneously by the Bradford assay and the modified empirical formula, as shown in supplementary Table S2. For the Bradford assay, a standard curve with a good linear relationship was first obtained with* Mtu*P49 as the standard, and the protein concentration in *Mtu*P49-CTG complexes was calculated as 0.222 ± 0.008 mg/mL. On the other hand, the formula (Eq. 1) was modified as “*C*_mg/mL_ = 1.340*A*_280_ − 0.636*A*_260_” for protein quantitation, and the protein concentration was calculated as 0.227 ± 0.010 mg/mL. These results indicated that both of these methods are satisfactory for quantifying protein concentration in *Mtu*P49–CTG complexes, then, the concentration of *Mtu*P49–CTG complex can be deduced.

## DISCUSSION

The present study showed that the Bradford assay and the UV-derived formula-based method were capable to accurately quantify proteins in nucleoprotein complexes. The basic mechanism of the Bradford assay is the binding between Coomassie brilliant blue G250 dye and particular amino acid residues within the proteins, such as arginine, histidine, phenylalanine, tryptophan, and tyrosine, as well as the hydrophobic interactions among them (Goldring 2015; Noble 2014). This means errors will be resulted from the difference in amino acid composition between the standard and the sample. Therefore, to achieve accurate quantitative analysis, the pure target protein should be taken as the standard, rather than the BSA protein (Goldring 2015; Noble 2014).

Except for the Bradford assay, the UV method is another approach for protein quantitation. The amino acids tryptophan (λ_max_ = 279.8 nm, *ε* = 5.6 L/(g·cm)), tyrosine (λ_max_ = 274.6 nm, *ε* = 1.42 L/(g·cm)) and phenylalanine (λ_max_ = 257 nm, *ε =* 0.197 L/(g·cm)) were believed to be the contributors to A_280_ (Goldring 2015; Noble 2014). Therefore, the UV method is not dependent on a standard, allowing to directly quantify proteins. However, this method is not appropriate for quantifying proteins in nucleoprotein complexes, owing to the interference of nucleic acid (Goldring 2015; Noble 2014). The formula “*C*_mg/mL_ = 1.55*A*_280_ – 0.76*A*_260_” was thus developed to correct the noisy signal of nucleic acid in a protein solution (Goldring 2015). But quantifying proteins in nucleoprotein complexes by this method was somewhat limited. After the modification of the two coefficients *K*_280_ and *K*_260_ using two paired values of A_260_ and A_280_ corresponding to samples with different N/Ps, the protein concentration in such complexes could be measured more accurately. It should be noted that, for each nucleoprotein complex, the empirical formula should be modified specifically. In actual applications, just the paired A_260_ and A_280_ values of two samples with different N/Ps are required, for example, pure protein and the one with large amounts of nucleic acids (N/Ps = 1.5–3).

In conclusion, the present study applied Bradford assay and the UV-derived formula-based method for the quantitative analysis of nucleoprotein complexes, which may make contributions to explore the interactions between proteins and nucleic acids at the molecular level.

## Conflict of interest

Jiang Chen, Hao Luo, Mei Tao, Zhongchuan Liu and Ganggang Wang declare that they have no conflict of interest.
